# Deep Learning-Based Mental Health Model on Primary and Secondary School Students' Quality Cultivation

**DOI:** 10.1155/2022/7842304

**Published:** 2022-07-06

**Authors:** Shuang Li, Yu Liu

**Affiliations:** Department of Psychology, Guizhou Minzu University, Guiyang 550025, Guizhou, China

## Abstract

The purpose was to timely identify the mental disorders (MDs) of students receiving primary and secondary education (PSE) (PSE students) and improve their mental quality. Firstly, this work analyzes the research status of the mental health model (MHM) and the main contents of PSE student-oriented mental health quality cultivation under deep learning (DL). Secondly, an MHM is implemented based on big data technology (BDT) and the convolutional neural network (CNN). Simultaneously, the long short-term memory (LSTM) is introduced to optimize the proposed MHM. Finally, the performance of the MHM before and after optimization is evaluated, and the PSE student-oriented mental health quality training strategy based on the proposed MHM is offered. The results show that the accuracy curve is higher than the recall curve in all classification algorithms. The maximum recall rate is 0.58, and the minimum accuracy rate is 0.62. The decision tree (DT) algorithm has the best comprehensive performance among the five different classification algorithms, with accuracy of 0.68, recall rate of 0.58, and *F*1-measure of 0.69. Thus, the DT algorithm is selected as the classifier. The proposed MHM can identify 56% of students with MDs before optimization. After optimization, the accuracy is improved by 0.03. The recall rate is improved by 0.19, the *F*1-measure is improved by 0.05, and 75% of students with MDs can be identified. Diverse behavior data can improve the recognition effect of students' MDs. Meanwhile, from the 60th iteration, the mode accuracy and loss tend to be stable. By comparison, batch_size has little influence on the experimental results. The number of convolution kernels of the first convolution layer has little influence. The proposed MHM based on DL and CNN will indirectly improve the mental health quality of PSE students. The research provides a reference for cultivating the mental health quality of PSE students.

## 1. Introduction

The purpose was to timely recognize and predict the mental disorders (MDs) of students receiving primary and secondary education (PSE) (hereafter PSE students) and improve their mental health. With the rapid development of China's economy, the spectrum of residents' diseases has expanded, with more and more people experiencing MDs [1]. MD has become an important public health threat in China [2]. In particular, mental health assessment (MHA) can be accomplished through both an individual's internal feelings and external manifest. Internally, MHA indexes involve the psychological quality. Externally, MHA covers social adaptability [3]. Thus, studying MHA is necessary to improve people's health awareness. PSE students have also become the victims as MDs further spread, especially among young people [4]. Mental health has gradually become an important factor affecting students' physical and mental development. The optimization of students' psychological quality and improving their mental health level has become one of the focuses of current education [5]. Mental health counseling (MHC) for PSE students is needed for the healthy growth of students and the main content of mental health education [6]. Accordingly, the mental health of PSE students will be studied.

MDs are seriously harmful to individuals, worsening their social adaptability and threatening their physical health. For families, MDs will induce psychological burden and raise the economic burden. For society, MDs will disrupt public order, consume social resources, and aggravate the social expenditure on disease. Among all disease hazards, the negative impact of MDs accounts for 37%. However, due to the lack of mental health knowledge and discriminatory attitude towards people with an MD, many people with MDs refuse to seek professional help. The PSE students' MDs are divided into many aspects, including aggressive behavior, lying, truancy, stealing away, Internet addiction, and attention deficit hyperactivity disorder (ADHD) [7]. At present, the school of mental health education mainly offers MHC measures [8]. Schools can set up MHC courses and integrate MHC and subject teaching to create a healthy campus culture. Meanwhile, establishing an MHC room or an MHC network integrating society, family, and school encourages the healthy development of MHC to work for students under PSE [9]. MHC must be designed to improve both students' and teachers' mental health, establish students' mental health files, and build a comprehensive mental health network [10]. Traditional MHC is based on big data technology (BDT). It has achieved some results. Nevertheless, mainly relying on manual work, traditional MHC faces the problems of incomplete mental health education and limited MD prevention ability [11]. The development of artificial intelligent (AI) technology provides the basis for the smart mental health education models. Neural network (NN) has a certain predictive ability and is widely used in MHA [12]. Based on the above analysis, this work introduces the deep learning (DL) technology to build an MHM. In particular, the relationship between the MHM and the mental quality training of PSE students will be discussed.

The purpose was to strengthen the timely detection of MDs of PSE students and improve their mental health. The DL-based MHM will effectively predict the MDs of PSE students and provide improvement direction for students' mental health. Cultivating mentally healthy students is essential for quality education because mental health is the foundation for other qualities. Thus, mental health improvement is key to uplifting students' overall quality. MSE students are the hope and future constructor of socialist China. Their mental health education is of particular significance. To this end, this work first analyzes the current situation of PSE students' mental health education. It then constructs a PSE student-oriented MHM based on DL. The proposed MHM is improved, and the questionnaire survey (QS) method collects PSE students' mental health status. PSE students' mental health is analyzed using the proposed MHM based on the QS results. The innovation of this work is to propose an improved convolutional neural network (CNN) based on the DeepPsy model. The proposed DeepPsy model includes two functional modules: student online trajectory extraction module based on CNN and long short-term memory (LSTM) network; data source extraction module based on fully connected neural network (FCNN). The research provides an improvement direction for developing PSE-oriented mental health education.

## 2. Materials and Methods

### 2.1. Research Status of MHM

With the social progress, more and more people pay attention to mental health, and studies are enriching on MHMs. The research of MHM plays a key role in group mental analysis. Mental characteristics are often reflected in the individual's work, rest rules, and daily behaviors. Therefore, scholars have begun to analyze the individual's mental health status from daily behavior data in the current research. Studies have shown that individuals' online behaviors affect their mental health [13]. Shafiei et al. clarified that search behavior could be used for personality recognition by studying its relationship with personality characteristics [14]. Chandra et al. designed an algorithm for predicting MDs based on Internet usage behavior and scored by Symptom Check List (SCL)-90 [15]. Shahid et al. observed facial expressions in users' online information and found that users with happy emotions spend more time surfing online [16]. Bory et al. designed a new model for detecting depression based on time-frequency analysis of network behavior and achieved good prediction results [17].

Levakov et al. used shared posts on social networking sites to predict the severity of users' MDs in the future [18]. Marzouk et al. regard mental quality as an individual's ability to integrate social expectations and self-satisfaction [19]. Kim et al. believed that mental quality was based on heredity and influenced the social environment, education, and personal efforts [20]. Jin et al. considered that mental quality was based on mental conditions, internalizing the environmental stimuli into stable and basic individual traits [21]. Based on previous research, this work defines mental quality as the individual's relatively stable mental health state in a certain spectrum under the influence of acquired and genetic environmental factors. It believes that mental quality includes genetic heredity and acquired education.

Under the current network technology, the research on individual mental health mostly starts from network behavior analysis. However, these studies still have some deficiencies. Most studies use QS to obtain labels, which may be inaccurate. Besides, previous studies are mostly based on the relationship between a single online behavior and mental health as the basis for analysis, and it is difficult to reflect on the diverse school behaviors of students. Due to the universality of PSE students' online behavior, Internet behavior will also be used as the starting point to track students with MDs. Multi-source data are used, including Internet access data and grade data.

### 2.2. Contents of Cultivating Mental Health Quality of PSE Students

**Figure 1:**
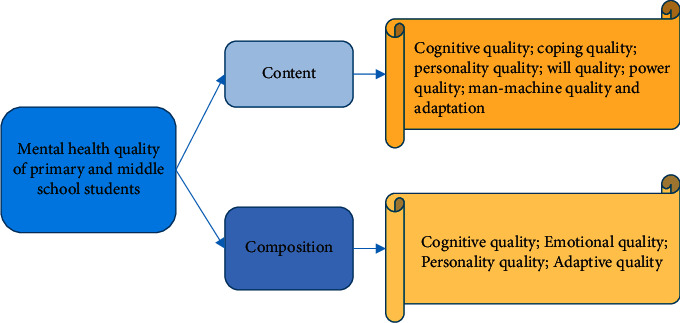
Connotation of mental health quality of PSE students.

The discussed mental health quality is the external manifestation and extension of mental health. The mental health quality requirements of PSE students are somewhat different from adults. Based on the content of the literature, the basic content of the current PSE students' mental health quality is summarized in Figure 1 [22].

In Figure 1, the mental health quality of PSE students includes cognitive quality, personality quality, coping quality, and will quality. The components include cognitive, emotional, personality, and adaptive quality. The following will build an MHM based on the content of the mental health quality of PSE students.

### 2.3. Construction of MHM Based on BDT

**Figure 2:**
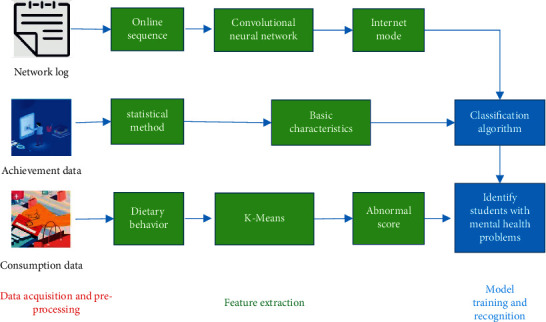
Overall framework of the MHM.

The overall framework of the MHM for PSE students is illustrated in Figure 2 [23].

In Figure 2, the overall framework of the MHM for PSE students is mainly divided into model training and recognition, feature extraction, and data acquisition and preprocessing.

The number of participants in this experiment is 210, including 70 primary school students, 70 junior high school students, and 70 high school students, randomly selected, with balanced gender control. The data acquisition method applies to relevant departments, recruits students uniformly, and collects various behavioral data of PSE students, including consumption data, network logs, historical performance data, and mental state data. In data preprocessing, noise data are removed for consumption data, and missing values are filled in grade data. Missing credits are calculated by the following equations:

(1) $$\begin{array}{c} F_{i}=\frac{\left({\text{Score}}_{i}-50\right)}{10}. \end{array}$$

(2) $$\begin{array}{c} Q_{i}={\text{Credit}}_{i}*{\text{GPA}}_{i}. \end{array}$$

In equations (1) and (2), *i* represents the course program number, *F*_*i*_ is the grade point, Score_*i*_ denotes the grade, Credit_*i*_ means the credit, and *Q*_*i*_ means the grade point average (GPA).

The GPA is also calculated as follows:

(3) $$\begin{array}{c} F_{\text{avg}}=\frac{\sum_{i=1}^{n}Q_{i}}{\sum_{i=1}^{n}{\text{Score}}_{i}}. \end{array}$$

The mental state data come from the school's psychological curriculum QS. After approval by the mental center and the informed consent of the students, the student's mental state table is obtained. The table includes the student's gender, grade, class, update time, and attention level. [24]. There are three levels of concern: severe MDs, moderate MDs, and mild MDs.

The feature extraction will mainly use the abnormal consumption score to find students prone to MDs. Studies have demonstrated that patients with MDs will have eating disorders [25]. The PSE students in the research area are all boarding, providing certain convenience for the research. By combining equations (4) and (5), the entropy of students' three meals a day is calculated, which will show the regularity of students' diet.

(4) $$\begin{array}{c} P\left(T=t_{i}\right)=\frac{n\left(t_{i}\right)}{\sum_{i=1}^{n}n\left(t_{i}\right)}, \end{array}$$

(5) $$\begin{array}{c} E=-\sum_{i=1}^{n}P\left(T=t_{i}\right)\log\;P\left(T=t_{i}\right). \end{array}$$

In equations (4) and (5), *n* represents the number of time intervals. *T* refers to the time interval set. *P* means the probability. *n* expresses the frequency. *t* indicates the subset of the time interval set *T*, and *E* denotes the entropy value.

There are two groups with similar entropy, one is to go to the cafeteria to eat occasionally and the other is to go to the cafeteria to eat frequently. Thus, the K-means clustering (KMC) algorithm is introduced to distinguish these two groups. The common dining behaviors of students include good, poor, and few dining regularities. Thus, *k* is set to 3. To discover students with abnormal diets, assume that the farther the students are from the cluster, the higher the abnormal data score is [26]. The student's abnormal score is calculated by the following equation:

(6) $$\begin{array}{c} M=D\left(n,c_{i}\right)*\left(1-\frac{\left|c_{i}\right|}{\left|C\right|}\right). \end{array}$$

In equation (6), *D* means the distance, *C* denotes all students, *c*_*i*_ is the centroid, *n* is a certain student, and *M* is the final score.

**Figure 3:**
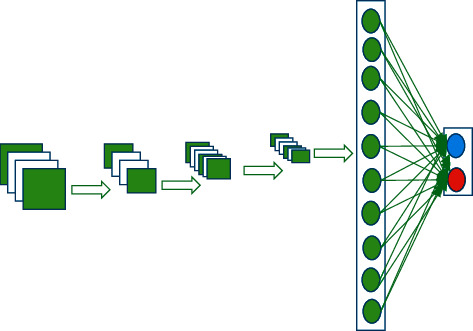
Framework of CNN.

Network feature extraction is performed based on CNN. The overall framework of the designed CNN is drawn in Figure 3 [27].

In Figure 3, the designed CNN has five layers; the first and the third layers are the convolution layer. The second and the fourth layers are pooling, and the fully connected layer is the fifth layer [28]. Different layer calculation is expressed by the following equation:

(7) $$\begin{array}{c} x_{j}^{l}=f\left(\sum_{i\in M_{j}}x_{i}^{l-1}*k_{\text{ij}}^{l}+b_{j}^{l}\right). \end{array}$$

In equation (7), *x*_*j*_^*l*^ is the feature map of the *j*th convolution kernel of the *l*th layer. *k* represents the convolution kernel, *b* expresses the bias, and *f* shows the activation function.

(8) $$\begin{array}{c} {\hat{x}}_{j}^{l}=f\left(w_{j}^{l}\right)\text{down}\left(x_{j}^{l}\right)+b_{j}^{l}. \end{array}$$

In equation (8), *w* represents a weight matrix, and down is a downsampling function.

(9) $$\begin{array}{c} s^{\text{pat}}=f\left(w^{\left(5\right)}*x^{4}+b^{\left(5\right)}\right). \end{array}$$

In equation (9), *s* is the final output of Internet mode.

The students' insomnia status is also counted, and the specific calculation is shown as follows:

(10) $$\begin{array}{c} P_{i}=\frac{t_{i}}{T}. \end{array}$$

In equation (10), *P*_*i*_ is the probability of insomnia, *i* refers to the student, and *T* means the number of days.

Five common classification algorithms are selected in this model building, and the most suitable classification algorithm is selected as the classifier. The data are divided into a test set, validation set, and training set based on the previous data collection. The performance evaluation indicators of the classifier are accuracy, precision, recall rate, *F*1-measure, and area under the curve (AUC) [29].

### 2.4. Optimization of MHM by DeepPsy

**Figure 4:**
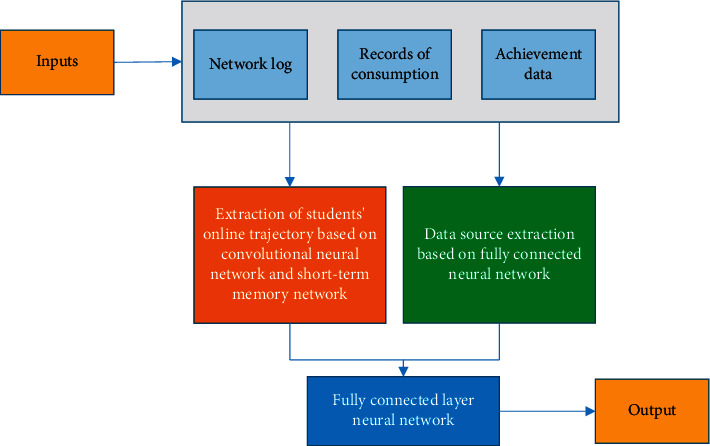
DeepPsy model.

Here, the proposed CNN model is optimized to improve its recognition efficiency [30] using the DeepPsy model. The DeepPsy model includes the students' online trajectories extraction module based on CNN and LSTM and the data source extraction module based on the FCNN, as detailed in Figure 4.

In Figure 4, the DeepPsy model connects the outputs of the two modules and finally uses an FCNN. All data in the model are connected. The direction propagation loss affects both modules simultaneously in model training.

After optimization, CNN has eight layers: the first and fourth layers are the convolution layer. The same convolution is used to ensure that the matrix dimension does not change after the convolution operation. The second and fifth layers are normalization layers that normalize the previous convolutional layer results within the batch. The fully connected layer is the seventh layer, combining the pooling layer's output features with the convolutional layer's output features. The dropout network layer can prevent overfitting during training, so the eighth layer is the dropout layer. The ninth layer is the hidden layer of the LSTM, and the tenth layer is the FCNN.

The parameter setting is as follows. The first and fourth layers use four 3 × 3 convolution kernels and 32 3 × 3 convolution kernels. The third and sixth layers use 2 × 2 pooling. The seventh layer uses 16 neurons, and the parameter of the eighth layer is 0.5. In *f* LSTM, the input vector is 64, and the number of neurons is 4. The number of the first fully connected neuron node of the basic feature module is 8, and the number of the second neuron node is 4. After the output values of the two modules are spliced, the input is a fully connected layer with four neuron nodes. The number of iterations in the model training is 60, the optimization algorithm adopts the Adam optimizer, the learning rate is 1*e* − 3, and the batch_size is 4.

## 3. Results and Discussion

### 3.1. Analysis of the Construction Results of MHM Based on BDT

**Figure 5:**
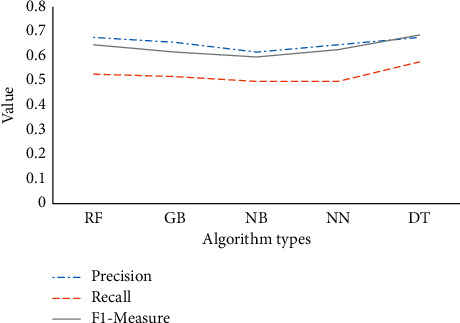
Results of different classification models.

This section introduces five machine learning models: random forest (RF), naive Bayes (NB), gradient boosting (GB), neural network (NN), and decision tree (DT). Then, it combines them with the proposed MHM based on BDT and analyzes the accuracy, recall rate, and *F*1-measure. The results are given in Figure 5.

Figure 5 shows that the accuracy curve is higher than the recall rate curve in all classification models. The recall rate is the largest at 0.58, and the accuracy rate is the smallest at 0.62. The results indicate that the proposed MHM can successfully identify a small sample set. The DT performs the best among the five classification models: accuracy, recall rate, and *F*1-measure of 0.68, 0.58, and 0.69. Based on this, the DT algorithm is chosen as the classifier. On the test dataset, the proposed MHM can obtain an accuracy of 0.68, a recall rate of 0.56, and an *F*1-measure of 0.67. It proves that the proposed MHM can identify 56% of the respondents with MDs. In the research on the performance of classification algorithms, the research results of Kumari and Sai also show that the classification effect of the DT algorithm is the best [31].

### 3.2. Analysis of Optimization Results of MHM by DeepPsy

**Figure 6:**
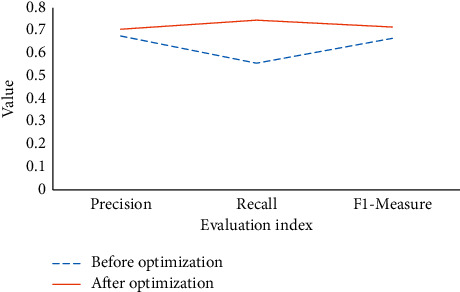
Comparison of model performance before and after optimization.

The performance of the proposed MHM before and after optimization by DeepPsy is compared in Figure 6.

Figure 6 manifests that after optimization, the proposed MHM's performance is better than before optimization. The accuracy is improved by 0.03, and the *F*1-measure is improved by 0.05. The recall rate represents the proportion of predicted students and directly reflects the model performance. The recall rate of the optimized model is increased by 19% to 0.75, indicating that the DeepPsy-optimized MHM can identify 75% of students with MDs. In the latest study on mental health recognition by Lee and Park, the recognition rate is 82%. Although the accuracy of this work is slightly lower than 82%, it is within the acceptable range [32].

**Figure 7:**
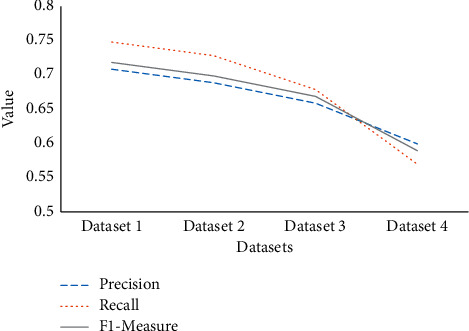
Comparison of results of different data features.

To further discuss the influence of features on the experimental results, the data sources are recombined. Dataset 1 is network features, performance features, and consumption features. Dataset 2 is performance features and consumption features, dataset 3 is network features and performance features, and dataset 4 is the performance features. Combined with the optimized model, the experimental results are obtained in Figure 7.

In Figure 7, the experimental results of dataset 1 are the best. The experimental results gradually deteriorate with the reduction in feature types, and dataset 4 has the largest decline. It is analyzed that the performance has a greater impact on the experimental results. Figure 7 denotes that diverse behavioral data can improve the identification of students' MDs. The research of Brad et al. also shows that the more data types are, the higher the efficiency of model recognition [33].

**Figure 8:**
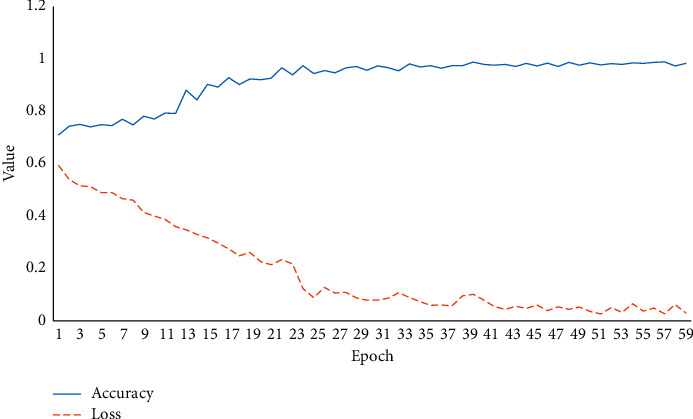
Robustness validation of the model.

The robustness of the DeepPsy-optimized MHM is verified, including the effect of the number of convolution kernels and batch_size. The results are revealed in Figure 8.

In Figure 8, as iteration continues, the model accuracy increases, and the loss decreases. Apparently, the model accuracy and loss tend to stabilize from the 60th iteration.

**Figure 9:**
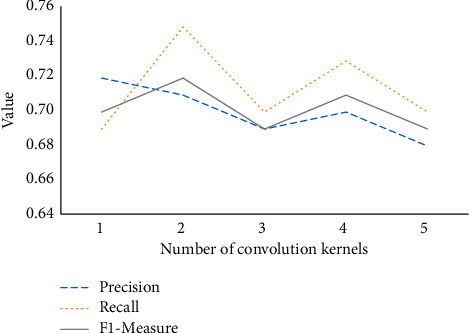
Effect of the number of convolution kernels on the model.

Figure 9 compares the model results of different convolution kernels in the first layer of CNN.

Figure 9 expresses that with five kinds of convolution kernels, the accuracy fluctuates about 4%, the recall rate fluctuates about 6%, and *F*1-measure fluctuates at 3%. Thus, the number of convolution kernels in the first convolutional layer has little effect on the experimental results.

**Figure 10:**
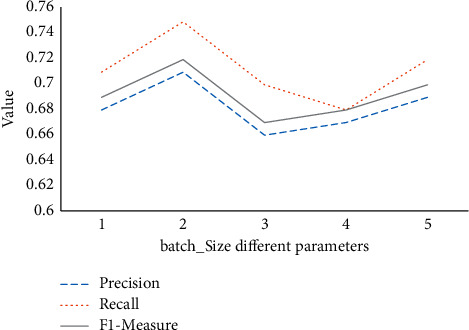
Effect of batch_size value on the model.

Under different batch_size parameters, the experimental results of the optimized model are compared in Figure 10.

In Figure 10, the accuracy oscillates at about 5%, the recall rate oscillates at about 7%, and the *F*1-measure oscillates at 5%. Compared with Figure 9, the difference in batch_size has a slightly greater impact on the experimental results, and the number of convolution kernels in the first convolutional layer has less impact on the experimental results.

### 3.3. The Influence of MHM on Mental Health Quality

**Figure 11:**
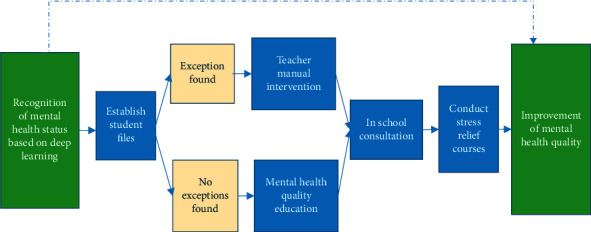
PSE student-oriented quality cultivation strategy based on the proposed MHM.

The PSE student-oriented quality cultivation strategy based on the proposed MHM is obtained combined with the previous content. The proposed MHM improves the time to identify students with MDs based on DL. Through MHM identification and recognition, artificial mental intervention can quickly follow up to improve the mental health quality of PSE students. The PSE student-oriented quality cultivation strategy based on the proposed MHM is portrayed in Figure 11.

In Figure 11, the proposed MHM based on the DL and DeepPsy-improved CNN will indirectly improve the mental health quality of PSE students. Teachers will provide special counseling once the proposed MHM finds students with MDs. In mental health quality cultivation, the creation of student files is particularly significant. The proposed MHM is based on student files and will also play a role in improving student files. Most of the research on students' mental health identification is completed based on students' files [34]. Students' files are of great significance for evaluating students' mental health status.

## 4. Conclusion

Under the background of the increasing number of young people with MDs, the PSE student-oriented mental health quality cultivation is studied. Firstly, the CNN in the DL technology is introduced to build an MHM. Secondly, DeepPsy is used to optimize the proposed MHM's recognition performance. Consequently, the PSE student-oriented quality cultivation strategy based on the proposed MHM is suggested following the previous research. The research puts forward an intelligent plan for cultivating the mental health quality of PSE students and provides an efficient MHM and technical support for the early intervention of students with MDs. Finally, there are still some deficiencies. The combination of the DL-based MHM and the quality training of PSE students is not deep enough. The recognition accuracy of the proposed MHM is about 75%, which cannot identify the severity of students with MDs. Therefore, future research will explore a deep integration mechanism for the PSE students' mental health quality cultivation and MHM. Meanwhile, a module to predict the severity of students' MDs will be added to design a more comprehensive and efficient mental health identification and quality training system.

## Data Availability

The datasets used and/or analyzed during this study are available from the corresponding author on reasonable request.
